# Effectiveness of multi-modal home-based videoconference interventions on sleep in older adults: study protocol for a randomized controlled trial

**DOI:** 10.3389/fpubh.2024.1326412

**Published:** 2024-04-15

**Authors:** Emma Milot, Stéphane Rehel, Antoine Langeard, Lucile Bigot, Florane Pasquier, Laura Matveeff, Antoine Gauthier, Nicolas Bessot, Gaëlle Quarck

**Affiliations:** ^1^Université de Caen Normandie, INSERM, COMETE U1075, CYCERON, CHU de Caen, Normandie Université, Caen, France; ^2^MOOVEN, Montpellier, France

**Keywords:** aging, physical exercise training, bright light exposure, galvanic vestibular stimulation, home-based intervention, polysomnography, sleep complaints, web-based

## Abstract

Aging is characterized by substantial changes in sleep architecture that negatively impact fitness, quality of life, mood, and cognitive functioning. Older adults often fail to reach the recommended level of physical activity to prevent the age-related decline in sleep function, partly because of geographical barriers. Implementing home-based interventions could surmount these obstacles, thereby encouraging older adults to stay active, with videoconference administration emerging as a promising solution. Increasing the availability of biological rhythms synchronizers, such as physical activity, light exposure, or vestibular stimulation, represents a viable non-pharmacological strategy for entraining circadian rhythms and potentially fortifying the sleep–wake cycle, thereby enhancing sleep in aging. This study aims to (1) assess the impact of remote physical exercise training and its combination with bright light exposure, and (2) investigate the specific contribution of galvanic vestibular stimulation, to sleep quality among healthy older adults with sleep complaints. One hundred healthy older adults aged 60–70 years with sleep complaints will be randomly allocated to one of four groups: a physical exercise training group (*n* = 25), a physical exercise training combined with bright light exposure group (*n* = 25), a galvanic vestibular stimulation group (*n* = 25) or a control group (i.e., health education) (*n* = 25). While physical exercise training and health education will be supervised via videoconference at home, bright light exposure (for the physical exercise training combined with bright light exposure group) and vestibular stimulation will be self-administered at home. Pre-and post-tests will be conducted to evaluate various parameters, including sleep (polysomnography, subjective questionnaires), circadian rhythms (actigraphy, temperature), fitness (physical: VO_2_ peak, muscular function; and motor: balance, and functional mobility), cognition (executive function, long-term memory), quality of life and mood (anxiety and depression). The findings will be anticipated to inform the development of recommendations and non-pharmaceutical preventive strategies for enhancing sleep quality in older adults, potentially leading to improvements in fitness, cognition, quality of life, and mood throughout aging.

## 1 Introduction

Aging induces changes in various physiological functions, including shifts in sleep patterns (1–3). Older adults often report difficulties in falling and staying asleep, spending increased time awake during the night, and experiencing daytime sleepiness (3–5). The decline in sleep quality with age also significantly impacts the quality of life (QoL), defined as “the degree of need and satisfaction within the physical, psychological, social, and environmental domains” (6). Evidence indicates that impaired sleep quality is closely associated with adverse effects on physical and psychological well-being, as well as limitations in daily activities and cognitive functions (7). Thus, enhancing sleep quality in older adults becomes a crucial objective for maintaining QoL.

The sleep/wake cycle is influenced by both endogenous and exogenous components. The endogenous component corresponds to our central biological clock, located in the suprachiasmatic nuclei of the hypothalamus (8, 9). This autonomous system ensures the genesis, maintenance, and control of biological rhythms, such as body temperature or cortisol levels, which are involved in glucose homeostasis. The exogenous component refers to the external synchronizers in our environment that contribute to entraining biological rhythms over a given period. Synchronizers can either modify the phases (phase shifts) or synchronize the circadian rhythms (10). In humans, the most powerful synchronizer for the biological clock is the 24-h light/dark cycle (11, 12). The impact of light on the circadian clock depends on the timing of light exposure: early morning light exposure advances the clock, while evening and night light exposure delays it (13, 14). Previous studies have demonstrated that bright light exposure helps synchronize circadian rhythms and improve QoL, sleep, and cognitive parameters (15–17). Another non-photic synchronizer identified in the literature is physical activity (18, 19). Previous studies have reported that physical exercise training in individuals over 60 years helps synchronize endogenous circadian rhythms, supporting the idea that non-photic stimuli also act as synchronizers for the human circadian clock (20). Additionally, exercise has been associated with reductions in daytime sleepiness and improvements in sleep quality (21, 22). Exercise has also been shown to reduce the use of hypnotics in this population, which is significant given the adverse side effects of these drugs on cognition, executive function, and mobility (23, 24). Additionally, maintaining moderate physical activity for 25–65 min per day can eliminate the risk of mortality associated with sleep disorders (25). However, the timing of exercise can be crucial for enhancing sleep quality, yet a limited number of studies on this subject involving older adults have been conducted (26). Acute exercise in the morning has been shown to improve nocturnal sleep quality in older adults with difficulty initiating sleep, as assessed with polysomnography (27), while acute vigorous exercise ending ≤1 h before bedtime affects sleep quality and awakenings compared to a control group (no exercise) (26). To our knowledge, only one study has investigated the effects of chronic exercise in the morning or evening (for 14 days) in older adults using polysomnography, and it reported no objective changes in sleep (28). Finally, exercise in older adults is also of interest because of its numerous positive effects on QoL, physical condition, and cognition (29–32). While bright light exposure and exercise are essential for the synchronization of circadian rhythms and significantly contribute to overall sleep quality, the effects of combined light and exercise exposures on sleep quality are not yet well known.

Unfortunately, one in three older adults fails to meet the recommended levels of physical activity (33), resulting in missed health benefits (33). Giné-Garriga et al. (34) showed that older adults allocate 78.8% of their waking time to sedentary behavior, 18.6% to light-intensity physical activities, and only 2.6% to moderate-to-vigorous physical activities. Seniors often cite barriers such as the cost of sports sessions or the distance between home and sports facilities (35, 36). Home-based remote supervised interventions could overcome these limitations and improve overall health (37–39). Videoconferencing technology can facilitate interactions between the participants and the physical trainers, providing a pragmatic alternative to face-to-face home visits by physical trainers (37–39). Several studies have demonstrated that digital platforms exhibit high rates of adherence, compliance, and satisfaction among older participants (37, 40). Additionally, videoconferencing has been shown to achieve the same benefits as face-to-face programs (41), making it an optimal solution. To our knowledge, this study is the first to evaluate the effects of various home-based interventions using videoconference and a digital monitoring platform on sleep quality in healthy older adults with sleep complaints.

All motor activities require the integration of sensory information. During exercise, the brain continuously processes sensory input to maintain balance, head position, and spatial orientation. This sensory information is handled by the vestibular system, located within the inner ear. Recent studies conducted in both animals and humans have suggested a link between the vestibular system and circadian rhythms (42, 43), suggesting that vestibular stimulation could serve as a synchronizer with potential positive impacts on sleep. Only a few studies have investigated the effects of vestibular stimulation on sleep. These studies have used vestibular stimulations induced by rocking movements or galvanic currents (i.e., a non-invasive method that stimulates the vestibular nerve using a direct low-intensity electrical current) (44–46). Polysomnography, a gold standard method (47), was used to record sleep in these studies. Research on rocking movements demonstrated improvements in sleep/wake transitions and alterations in electroencephalographic activity during nights or naps experienced on a moving bed compared to a stationary one (44, 45). Only one study, employing a specific model involving an advance in bedtime by 4 h, has explored the effects of galvanic vestibular stimulation on sleep in a specific population (insomniacs), which revealed a reduction in persistent sleep latency (46). However, the effects of galvanic vestibular stimulation on the sleep of older adults under ecological conditions (without phase advance) remain to be evaluated. Galvanic vestibular stimulation emerges as an innovative and easily administered technique, presenting an interesting prospect for resynchronizing circadian rhythms and improving sleep.

The aims of this study are (1) to evaluate the effects of remote physical exercise training and the combined effects of remote physical exercise training and bright light exposure on sleep quality in healthy older adults with sleep complaints, and (2) to study the specific contribution of galvanic vestibular stimulation on sleep quality in the same population. By reinforcing circadian rhythms, the various interventions should improve sleep quality, physical and motor fitness, cognition, and, consequently QoL.

## 2 Materials and methods

### 2.1 Study design

The study is a randomized controlled trial with four parallel groups: a Physical Exercise Training group (PET), a PET combined with Bright Light Exposure group (PET + BLE), a Galvanic Vestibular Stimulation group (GVS), and a control group [Health Education (HE)]. All participants will be provided written informed consent before their inclusion. The protocol has been approved by the local ethical committee (Comité de Protection des Personnes, CPP Nord Ouest III, Caen) in compliance with French regulations (trial registration number: IDRCB: 20206A01578-31). The researchers will provide participants with detailed written and oral information about the trial, ensuring voluntary participation and emphasizing that participants may withdraw at any time.

### 2.2 Participant

Participants will be recruited through advertisements in the local newspapers and media reports. One hundred healthy retired participants, both male and female, aged between 60 and 70 years and independently living in Normandy, will be enrolled. Exclusion criteria will include: not having computing equipment (computer, web connection and webcam), signs of cognitive impairment reflected by a Mini-Mental State Examination (MMSE) score below 26/30 (48), no sleep complaint reflected by a Pittsburgh Sleep Quality Index (PSQI) score > 5 (49), extreme circadian typology reflected by the Morningness-Eveningness Questionnaire (MEQ) (50) score below 31 or above 69, uncontrolled psychiatric, vestibular, neurologic, endocrine or cardiovascular affections, uncorrected hearing and/or visual impairment, psychotropic or bradicardizing medical treatments, alcohol, drug or caffeine abuse, and recent hospitalization (<30 days), all to be assessed by medical evaluations and screenings. Participants ranking moderately or highly active according to the International Physical Activity Questionnaires (IPAQ) (51) will be excluded. The ability to engage in moderate to vigorous physical activity will be assessed by a medical examination and cardiac screening during a maximal incremental test (VO_2_ peak) on a cycle-ergometer (detailed below), supervised by cardiologists at the sports medicine department of the local hospital of CHU in Caen. Complete inclusion and exclusion criteria are listed in Table 1. Eligible participants will be allocated to one of four groups using simple randomization from a computer-generated numbered sequence with 25 blocks of 4, to be conducted by researchers. Participants will be assigned a number in the order of their registration inclusion in the study.

**Table 1:** Inclusion and exclusion criteria.

Inclusion criteria	Exclusion criteria
Participant ≥60 ≤ 70 y.o Retired for ≥12 months Good health condition Living in Normandy Sedentary behavior Sleep complaints reflected by a PSQI score > 5 Computing equipment Satisfactory cardiac screening	Cognition <26/30 on the MMSE IPAQ = moderately and highly active Circadian typology = extreme chronotype Sign of cognitive impairment Uncontrolled psychiatric, vestibular, neurologic, endocrine or cardiovascular affections Uncorrected hearing and/or visual impairment Psychotropic or bradicardizing medical treatments Recent hospitalization (<30 days) Alcohol, drug or caffeine abuse

### 2.3 Assessments

**Figure 1:**
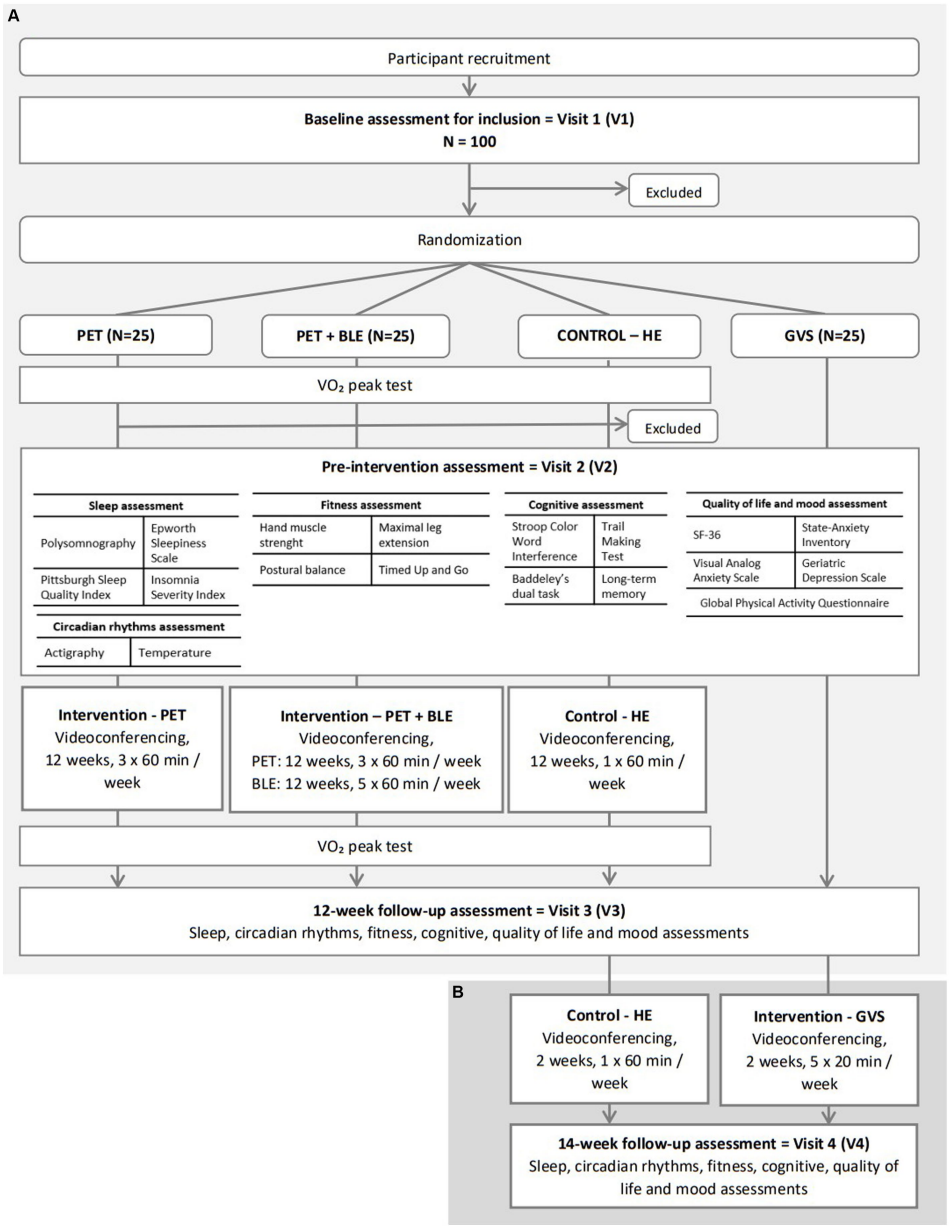
Participant’s flow chart. **(A)** The first part of participant’s flow chart: PET and PET+BLE interventions; **(B)** the second part of participant’s flow chart: GVS intervention. PET, Physical Exercise Training; BLE, Bright Light Exposure; HE, Health Education; GVS, Galvanic Vestibular Stimulation.

Inclusion and randomization will occur at the baseline assessment (visit 1) and will involve the completion of pre-inclusion questionnaires. A pre-intervention visit will take place before the start of the intervention (visit 2) and a 12-week follow-up assessment will occur at the end of the 12-week intervention (visit 3) (Figure 1A). A final assessment will be performed after completion of the 14-week intervention, but only for control and GVS groups (visit 4) (Figure 1B). According to the literature, a typical GVS protocol is short and does not exceed 2 weeks (52). Therefore, the GVS protocol will be shorter than PET and BLE interventions to ensure participant feasibility and acceptability. The two weeks of vestibular stimulation will be scheduled after the 12-week follow-up to ensure consistent design (Figure 1B). The control group will be compared to the PET and PET + BLE group, but also to the GVS group.

Sleep, circadian rhythms, physical and motor fitness, cognitive, QoL, and mood measurements will be assessed during visits 2, 3, and 4, by researchers in our laboratory. The VO_2_ peak test will be performed during visits 2 and 3 only for PET, PET + BLE, and control groups (not for the GVS group, which will not engage in physical exercise) and will allow the exclusion of participants with cardiac disorders. These tests will be conducted by cardiologists at the sports medicine departments of the local hospital of CHU in Caen. The content of all assessments is described in Figure 1.

#### 2.3.1 Sleep assessment

Participants will undergo a polysomnography (PSG) recording at home using a portable device (Embletta^®^ MPR Sleep System, NATUS, Orlando, United States). Ten electrodes will be placed over the scalp according to the international 10–20 system: frontal (F3 | F4), central (C3 | C4 | Cz), occipital (O1 | O2), ground (Cz | Fz), and a bi-mastoid reference (A1 | A2), with impedance kept below 15 kΩ. This montage will also include an electrooculogram (EOG), an electrocardiogram (ECG), and a chin electromyogram (EMG). Respiratory movements, airflow, and oxygen saturation will be recorded with abdominal and thoracic belts, nasal and oral thermistors, and finger pulse oximetry. The Electroencephalogram (EEG) signal will be digitized at a sampling rate of 256 Hz. High-pass and low-pass filters will be applied, respectively, at 0.3 Hz and 35 Hz. PSG recordings will be manually scored in 30-s epochs following the recommended standard criteria of the American Academy of Sleep Medicine (53) using RemLogic software. Sleep analyses will be conducted to calculate standard sleep parameters, including total sleep time (TST, min), sleep onset latency (SOL, min), wake after sleep onset (WASO, min), sleep efficiency (SE, %), percentage of time spent in each sleep stage (S1, S2, Slow-Wave Sleep, and Rapid-Eye Movement sleep, %), and apnea-hypopnea index (AHI). Sleep apnea will be defined by a ≥ 90% drop of nasal pressure for at least 10 s, whereas sleep hypopnea will be characterized by a ≥ 30% drop of nasal pressure for a minimum of 10 s, associated with an arousal or an oxygen desaturation of ≥3% (53).

Sleep quality and quantity will be evaluated using validated self-report questionnaires. Sleep quality and disturbances over the last month will be evaluated using the Pittsburgh Sleep Quality Index (PSQI) (49) through different parameters, including work, sleep habits, sleep disturbances, subjective sleep quality, the use of sleep medication, and trouble staying awake during the day. Daytime sleepiness will be assessed using the Epworth Sleepiness Scale (ESS) (54) and insomnia over the last month will be assessed with the Insomnia Severity Index (ISI) (55). Participants will complete a sleep diary to subjectively assess sleep quality and duration, including hours of bedtime, sleep quality, number, and duration of nocturnal awakenings and naps.

#### 2.3.2 Circadian rhythms assessments

The sleep–wake cycle and body temperature, both strong circadian markers, will be evaluated using actigraphy and tympanic temperature measures.

The sleep–wake cycle will be continuously recorded over a 7-day using the Motion Watch 8 wrist-worn triaxial actigraph (CamNTech Ltd., Cambridge, United Kingdom). It is a highly reliable tool that has been validated for older adults (56, 57) and in the participant’s home living conditions (58). The Motion Watch Motion Ware software (Cambridge Neurotechnology Ltd., Cambridge England) combined with this actigraph will provide measures of TST (min) SOL (min); WASO (min); SE (%); sleep fragmentation index (SFI); and non-parametric circadian rhythm analysis [average of the Least active 5-h periods (L5, counts); average of the most active 10-h periods (M10, counts); Inter-daily Stability (IS); Intra-daily stability (IV); and Relative Amplitude (RA)].

Tympanic body temperature will be measured using a clinical thermometer (Braun ThermoScan^®^ 3). The temperature (in degrees Celsius °C) will be recorded over a 1 day, during the actigraphy period, with two readings for the right ear and two readings for the left ear. Participants will take 7 temperature readings over 24 h (6:00 am, 9:00 am, 12:00 pm, 3:00 pm, 6:00 pm, 9:00 pm, 12:00 am). Before each tympanic temperature measurement, participants will start with a 15-min rest on their back, without eating or drinking (59).

#### 2.3.3 Physical fitness assessment

Muscular strength and cardiovascular capacities will be tested to assess physical fitness.

The evaluation of maximal hand grip strength will be recorded using a hand dynamometer (Takei A5401 Hand Grip Dynamometer Digital, Takei Scientific Instruments Co., Japan). Three successive measurements will be collected in a standardized position: participants will be seated on a chair without armrests, clenching their hands around a dynamometric handle. Their hand will rest on their leg to relax their shoulders and avoid elbow movements. The force developed in isometry by the gripping force of the hand will be recorded in kilograms.

The evaluation of maximal leg extension power will be recorded using the Nottingham Power Rig (Medical Engineering Unit, University of Nottingham Medical School, Nottingham, United Kingdom), specifically designed for older adults (60). Eight successive measurements will be collected in a standardized position for the right leg only. Participants will adopt a seated position with arms bent over the torso and will perform a maximal foot push to a large foot pedal. The power developed at the maximal speed of the flywheel after the pedal push, measured in watts, is recorded (60).

Peak Oxygen uptake (VO_2_ peak) will be measured with a maximal incremental test on a bicycle ergometer (eBIKE COMFORT, General Electric HealthCare) under medical supervision. Participants will follow a 3-min warm-up at 30 watts and will be instructed to pedal for as long as possible while the load will increase by 20 watts every 2 min. Developed power, and O_2_ and CO_2_ flow rates will be recorded before the test (resting values), at each level (relative values), until the end of the test (maximal values), and during the following 3 min of passive recovery (recovery values). Heart rate (HR) and electrocardiogram will be continuously recorded to track possible cardiac irregularities and stop the protocol if necessary. Criteria for reaching a VO_2_ peak (61) will include achieving a plateau in heart rate and VO_2_ recordings, inability to follow the imposed pedaling velocity, or reaching a given resistance. The main recorded parameters will include maximum oxygen uptake (mL/min/kg), maximum aerobic power (W), maximum HR (bpm), and respiratory quotient measured during the test.

#### 2.3.4 Motor fitness assessment

Motor fitness will be assessed through tests of balance and functional mobility.

Postural static balance will be assessed using a standard static force platform (SPS system, Synapsys, Marseille, France), with dimensions of 50 × 50 × 17.5 cm. After removing their shoes, participants will be required to place their feet apart, forming an angle of 30°, in the middle of the platform. Two trials with eyes open (EO) and two trials with eyes closed (EC), each lasting 30 s, will be performed in a static position. For each condition, the sway area (mm^2^) and the Standard Deviation (mm) of the sway of the center of pressure will be analyzed along the Medial-Lateral (ML) and Anterior–Posterior (AP) axes. The Romberg quotient (RQ) will be calculated to evaluate the role of visual information in static balance, representing the ratio between the surface parameter of the center of pressure with EC to the one measured with the eyes open EO. Also, the maximal limit of the stability test will be recorded without time limitation (mm^2^). In this test, participants, with their eyes open, will swing as far as possible in all directions while keeping their bodies straight and without moving their feet or falling. The researcher will stop the test when the participant no longer improves their limits of stability.

Functional mobility will be assessed using the Timed Up and Go (TUG) test (62, 63). The performance time will start when the participant will begin to move to stand up and will end when the participant will sit down again. The participant will start by sitting on a chair with their feet on the floor and hands on their legs. At the “go” signal, they will stand up, walk 3 meters, turn around a cone, walk back, and sit down again. Two trials at a self-paced speed and two as fast as possible will be performed and measured in seconds.

#### 2.3.5 Cognitive assessment

Cognitive executive functions will be evaluated using tests from the GREFEX battery (64). Inhibition will be assessed using the Stroop Color-Word Interference test (65). Mental flexibility will be assessed using the Trail Making Test (TMT) (52). The dual-task performance will be assessed using Baddeley’s dual task (66). Long-term memory will be assessed with a computerized task based on the memory game principle (67). This task, adapted from Rasch et al. (67), will evaluate the consolidation of visuospatial learning during sleep and will involve memorizing 12 pairs of cards. Learning will be considered achieved when 67% of the responses will be correct (a total of 8 pairs out of 12). A recall will be proposed the next morning after the night’s polysomnography. The number of pairs of cards correctly retrieved after learning and after the night’s sleep will be collected to calculate a rate of improvement or forgetfulness during the night.

#### 2.3.6 Quality of life and mood assessment

QoL will be evaluated using the SF-36 Health Survey (68), which is based on eight physical and mental health domains: physical function, role-physical, bodily pain, general health perceptions, vitality, social functioning, role-emotional, and mental health. The Global Physical Activity Questionnaire (GPAQ) will evaluate participants’ physical activity through different behavioral domains, including work, transport, and leisure (69).

Mood symptoms, including anxiety and depression, will be assessed using the State–trait Anxiety Inventory (STAI state subscale and STAI trait subscale) (70), Visual Analog-Anxiety Scale (VAS) (71), and Geriatric Depression Scale (GDS) (72, 73).

### 2.4 Interventions

PET and HE interventions will be delivered via videoconferencing using a secure web-based platform named SAPATIC (Santé Activités Physiques Adaptées utilisant les Technologies de l’Information et de la Communication developed by Mooven, Montpellier, France). BLE and GVS interventions will involve remote monitoring using a web-based platform. All participants will be able to connect to the platform at home and will have access to the planning and follow-up of their interventions. All participants’ data will be hosted on an authorized health data host.

#### 2.4.1 Physical exercise training (PET)

PET, designed according to the World Health Organization (WHO) and the American College of Sports Medicine (ACSM) guidelines (74), will be supervised by qualified physical trainers. Participants will follow a 12-week home-based physical exercise training intervention with the objectives of improving muscular function and aerobic capacity and maintaining autonomy. The intervention will consist of two 60-min aerobic sessions per week via videoconferencing (with real-time coaching) and one 60-min self-guided strength training session per week (without real-time coaching), totaling 36 sessions. PET sessions will be scheduled in the early afternoon. Participants will be provided with a step bench for performing different exercises before starting the physical exercise training intervention. Heart rate monitoring (OnRythm 500^®^) will also be provided to each participant to monitor the intensity level of physical effort. The target heart rate will increase over the weeks, starting at 60% of their reserve heart rate (determined during the VO2 peak test) and reaching 75% at the end of the intervention. Perceived pain, fatigue, and well-being (visual analog scales) will be evaluated at each session (videoconferencing and self-guided sessions) before, during, and after sessions. Perceived exertion (Borg rating) will be evaluated after each session. This information will enable the professional to ensure that the work is performed at the targeted intensity while adapting to the participant’s tolerance.

The two collective videoconferencing sessions per week will consist of aerobic exercises (i.e., run/walk on the spot, jumping jacks, step, or dance). Each 60-min PET session will consist of a verbal contact sequence, warm-up exercises (5–10 min), aerobic exercises (40 min), a cool-down period (5–10 min), and finally, a verbal feedback sequence. The number of repetitions and exercise difficulty will gradually increase during the sessions, modulated according to the participant’s capacities at the time of the exercises. The physical trainer will progressively increase activity difficulty in duration, then frequency, and ultimately intensity only if the participant tolerates previous increases. The sessions will be limited to 5 participants, enabling the physical trainer to adapt the exercises to each individual.

The self-guided sessions will focus on resistance exercises to increase arm, leg, and whole-body muscle strength (i.e., series of ventral/back/side plank, wall sit, pumps, lunges, squats, or front/side raises). Participants will receive a booklet containing detailed instructions for each exercise. Core training sessions will last 40 min, with the remaining 10–15 min equally allocated to warm-up and cool-down. The booklet will provide variations for each exercise to increase difficulty.

#### 2.4.2 PET combined with bright light exposure (BLE)

Participants in the BLE group will receive the same remote exercise training intervention as the PET group. The PET intervention will be supplemented with 12 weeks of bright light exposure sessions at home. Each participant will be provided with a light lamp (DAYVIA^®^ 072, France) and will be instructed to self-administer light sessions 5 days a week (from Monday to Friday) for 1 h in the morning (between 8:00 am and 10:00 am). During the sessions, participants will face the 10,000-lux light box at a distance of 50 cm and 45° from the eyes, avoiding direct staring to minimize discomfort. The intervention will be remotely monitored on the SAPATIC digital platform, in specific: exposure days, exposure time, and remarks filled in by the participants.

#### 2.4.3 Galvanic vestibular stimulation (GVS)

Galvanic Vestibular Stimulation (GVS) is a non-invasive method used to stimulate the vestibular system. The GVS paradigm involves the bilateral bipolar mode, with the anode electrode placed on the mastoid bone behind one ear and the cathode electrode behind the other ear.

Bipolar galvanic stimulations will be delivered by the mini-CT portable box (Soterix Medical^®^, United States). Participants will self-administer five sessions per week at home (from Monday to Friday) over 2 weeks, each lasting 20 min. The intensity will be set to 1 mA, initiated by a 60-s ramp-up and terminated by a 60-s ramp-down (52). Each session will be scheduled at the same time in the morning.

The researcher will program the GVS device before the intervention, configuring it according to the specified parameters and generating 10 unique session codes for each participant, corresponding to the 10 stimulation sessions. Throughout the intervention, participants will be instructed to enter only the code assigned to the day’s session. The quality of skin-electrode contact will be systematically checked, and if the signal is deemed “poor,” the stimulation will not initiate. The electrodes will be pre-soaked in saline solution, to minimize cutaneous sensations. The first vestibular stimulation session will be conducted in the laboratory with the experimenter, after which participants will be guided to self-administer subsequent sessions at home. The intervention will be remotely monitored through the SAPATIC digital platform.

#### 2.4.4 Control group-health education (HE)

The participants in the active control group will attend a one-hour health education session each week for a total of 14 weeks. These videoconference sessions will cover topics such as the benefits of physical activity, healthy lifestyles (including sleep habits and dietary balance), and cardiovascular risk factors. The inclusion of an active control group will aim to mitigate any potential influence of social contact and motivational factors on performance differences between the groups.

### 2.5 Data collection and management

The participants to be included will be registered with an identification number, ensuring anonymity for all collected data. Trained staff will collect the data under the supervision of the principal investigator. The data will be stored on a separate hard disk and processed anonymously. Each identification code will correspond to a laboratory file, documenting test results from baseline, before, and after interventions. Documentation of each physical training session will be collected and saved on the secure web-based platform (SAPATIC^®^). The balance between benefits and risks is favorable, with minimal adverse events observed for PET (muscular pain or contractures; risk of cardiovascular problems; risk of falling), BLE (headache, eye strain, nausea), and GVS (nausea, dizziness, swaying sensation, metallic taste in the mouth). Any adverse events will be documented in a study monitoring register. To ensure data quality, a trained project assistant will review data entries and cross-check the digitized version once all training and assessments are completed.

According to the ethical registration, there will be no independent data safety monitoring committee for this study. The conduct and progress of the study will be continuously monitored by the principal investigators and scientists and will be audited by the sponsor at mid-study and the end of the study.

### 2.6 Sample size calculations

The sample size calculation has been performed using R statistical software (R Core Team). In order to detect relevant differences between groups in the changes from pre-to post-intervention, we determined a sample size sufficient to detect medium effect sizes (Cohen’s *f* = 0.4). Therefore, α = 0.05, and *p* = 0.90, were selected to favor clinically significant effect sizes. The sample size calculation is based on sleep quality as the main variable. A total of 24 participants per group are necessary to examine the hypothesis that interventions impact sleep quality. Two additional participants are included to compensate for possible drop-outs. Each of the four groups will consist of 25 participants, resulting in a total of 100 participants.

### 2.7 Statistical analyses

Data will be analyzed with R statistical software (R Core Team, Austria). The statistical analysis will determine the effects of the three different training interventions on sleep, fitness, cognitive, and quality of life variables compared to a control group. The analysis will include subjects who completed all the pre-and post-tests. If participants drop out or deviate from the intervention protocols, their data will not be used. Deviations will include non-eligibility, withdrawal of consent, missed test visits, and intervention protocol not completed. Data from the pre-test and post-test will be presented using descriptive statistics: mean, standard deviation, and percentage to describe the participants’ characteristics and performance on assessments. Comparisons within and between four groups at baseline and post-intervention will be done using repeated measures ANOVA, after checking for normality. Assessments will be considered as the dependent variables, and the group will serve as the independent variable. *Post hoc* analyses will be conducted when a significant main effect is observed using Tukey’s test to correct for multiple comparisons.

The initial ANOVA, comparing the PET, PET, and BLE, control group, will aim to assess the presence of intervention effects (pre/post). The hypothesis is that the combined PET and light interventions will result in improved outcomes in both groups compared to the control group across various domains, including physical and motor fitness, cognitive, QoL, circadian rhythms, and sleep variables.

The second ANOVA, comparing the GVS and control groups, will be designed to assess the existence of intervention effects (pre/post). The hypothesis is that the GVS intervention will lead to improvements in balance, cognition, QoL, circadian rhythms, and sleep variables compared to the control group.

## 3 Discussion

This study aims to demonstrate the positive impacts of non-pharmaceutical remote interventions (PET, the complementary contribution of BLE to PET, and the specific contribution of GVS) on sleep quality. The study also aims to demonstrate how these interventions can result in improvements in circadian rhythms, overall sleep, physical and motor fitness, cognition, QoL, and mood parameters.

Although the benefits of physical activity on individuals’ health are widely acknowledged (21, 22, 29–31), few studies have examined the efficacy of PET using videoconferencing on fitness, cognition, QoL, and mood parameters in healthy older adults (37–39) and none on sleep. Moreover, current literature supports the advantages of remote physical training conducted through videoconferencing (39, 41, 75, 76). Our primary aim is to determine whether this training modality also yields positive effects on sleep. The second aim is to determine whether the addition of remotely monitored self-administered BLE to videoconference physical exercise training can optimize sleep improvements. Studies exploring the impact of combining these two synchronizers are still rare, focusing primarily on quality of life and mood (77, 78), with none specifically addressing sleep.

Studies conducted in both animals and humans have suggested a connection between the vestibular system and circadian rhythm (42, 43). There has been limited rigorous research on the effect of GVS on sleep quality, particularly in cases of insomnia sleep disorders. Krystal et al. (46) showed, using polysomnography, that galvanic vestibular stimulation induces a reduction in sleep latency only in participants whose sleep latency was greater than or equal to 14 min. Goothy and McKeown (79) observed a significant decrease in the ISI scores in healthy adults, indicating improvements in subjective sleep parameters following the GVS protocol. These studies provide preliminary evidence of the specific contribution of GVS on sleep quality, but none have been conducted in healthy older adults with sleep complaints. Further research is needed to assess the role of vestibular stimulation as a non-photic stimulus in synchronizing circadian rhythms.

This study has several strengths. Firstly, videoconference interventions prove to be a feasible and efficient method, effectively overcoming various barriers to intervention in this population (36–40). Also, videoconference interventions enhance adherence and compliance by facilitating real-time interactions between physical trainers and participants (37–41, 75). Secondly, the vestibular stimulation is delivered using a portable device, potentially providing individuals with reduced mobility access to a circadian rhythm synchronizer by replicating natural head movements similar to those experienced during physical activities (80). Thirdly, sleep disorders are frequently associated with the use of benzodiazepine and other sedative-hypnotics, the use of which is not recommended or limited due to serious side effects such as impaired global cognition, executive function, and mobility (81, 82). Thus, exploring various non-pharmaceutical interventions represents a promising strategy for managing these disorders.

The first possible limitation of this study will be that some participants might be discouraged by the use of new technologies. However, nowadays, older adults are becoming more familiar with technology, and we anticipate that its use will not be a significant issue. Finally, some participants may be discouraged by the length of the protocol, but compensatory allowances are provided to encourage them to continue with the interventions.

To conclude, the findings of this study are expected to contribute to the development of recommendations and non-pharmaceutical preventive strategies aimed at enhancing sleep quality in older adults. Consequently, these interventions are expected to lead to improvements in physical and motor fitness, cognition, QoL, and mood as individuals age.

## 4 Ethics and dissemination

The protocol was drafted by the Declaration of Helsinki. It was then submitted to the French Health Authority, specifically the National Agency of the Safety of Medicines and Health (ANSM), for formal approval to conduct the study and to ensure compliance with local regulations by a duly constituted Ethics Committee. Approval was obtained from the Health Authorities (ANSM) and the “Nord-Ouest IV” Ethics Committee in France, by French regulations (ID-RCB number 2020-A01578-31). Any protocol modifications will be promptly communicated to relevant parties, including investigators and the ethics committee. Eligible participants will initially be briefed about the study by the researcher, followed by comprehensive written and oral explanations provided by the investigators. Participation will be entirely voluntary, and individuals can withdraw at any time while still receiving standard treatment. Written informed consent will be obtained from all participants before their inclusion in the study.

The results of this study will be disseminated to the scientific community through presentations and publications in peer-reviewed scientific journals, as well as other stakeholders.

## Ethics statement

The studies involving humans were approved by “Nord-Ouest IV” Ethics Committee and French Health Authorities (ANSM)(ID-RCB number 2020-A01578-31). Clinical Trials (ID: NCT05030389). The studies were conducted in accordance with the local legislation and institutional requirements. The participants provided their written informed consent to participate in this study.

## Author contributions

EM: Data curation, Formal analysis, Investigation, Project administration, Supervision, Visualization, Writing – original draft, Writing – review & editing. SR: Data curation, Formal analysis, Investigation, Project administration, Visualization, Writing – review & editing. AL: Data curation, Formal analysis, Investigation, Project administration, Supervision, Visualization, Writing – review & editing. LB: Conceptualization, Methodology, Writing – review & editing. FP: Conceptualization, Methodology, Writing – review & editing. LM: Investigation, Writing – original draft. AG: Conceptualization, Methodology, Writing – review & editing. NB: Conceptualization, Data curation, Formal analysis, Funding acquisition, Investigation¸ Methodology, Project administration, Supervision, Validation, Visualization, Writing – review & editing, Resources. GQ: Conceptualization, Data curation, Formal analysis, Funding acquisition, Investigation¸ Methodology, Project administration, Supervision, Validation, Visualization, Writing – review & editing, Resources.
